# Understanding the Impact of COVID-19 on the Utilization of Community Health Services: Evidence from Beijing in China

**DOI:** 10.3390/healthcare13070707

**Published:** 2025-03-23

**Authors:** Yuqing Zhang, Lele Li, Qiao Yu, Qi Li

**Affiliations:** 1 School of Public Policy and Management, Tsinghua University, Beijing 100084, China; 2 Asia Competitiveness Institute, Lee Kuan Yew School of Public Policy, National University of Singapore, 469C Bukit Timah Road, Singapore 259772, Singapore; 3 School of Labor and Human Resources, Renmin University of China, Beijing 100872, China; 4 School of Public Administration, Sichuan University, Chengdu 610052, China; 5 The 21st Century Development Research Institute, Tsinghua University, Beijing 100084, China; 6 Yuyuantan Community Health Service Center, Haidian District, Beijing 100142, China

**Keywords:** community health services, COVID-19, health services utilization, Anderson model, health economics, public health

## Abstract

**Background:** Primary healthcare (PHC) at the community level is essential to improving access to health services and reducing healthcare costs. However, compared to developed countries where PHC is well developed, PHC in developing countries is not as well developed. The worldwide prevalence of COVID-19 has put a tremendous strain on the healthcare systems of all countries. Interestingly, we found that the impact of COVID-19 on the global healthcare system has brought about a new opportunity for PHC in developing countries. **Methods:** Based on community-scale panel data from Beijing, China, in the period from 1 January 2018 to 30 June 2021, this paper employed the Anderson model to reveal the impact of COVID-19 on community health service utilization. Two-way fixed effects models and double-difference models were used to analyze the data for robustness. **Results:** The results show that COVID-19 significantly reduced visits to the Community Health Center, but meanwhile, it raised the cost of single visits. While the costs of treatment, together with medical materials, were significantly lower during the pandemic, COVID-19 has affected the cost control of family physicians, resulting in the cost of contracted patients being significantly lower than that of non-contracted patients. **Conclusions:** While the COVID-19 pandemic significantly reduced routine visits to established medical centers, it served as a catalyst for the increased utilization of community health services. If appropriate measures to contain COVID-19 were taken, it would be possible to extend the scope of community health services and strengthen the PHC system.

## 1. Background

Primary healthcare (PHC) in grassroots communities is crucially important to improving the accessibility of health services, promoting the health of the population, and reducing healthcare costs [1,2,3]. Thus, improving the utilization of community health services for patients plays a key role in building hierarchical treatment, optimizing the allocation of medical resources, and promoting the sustainable development of health insurance funds [4,5,6]. Unlike developed countries where the PHC has been well developed, community health services in China are still in the promotion stage, and “hierarchic healthcare” is more often mentioned than “primary healthcare” [7,8].

Developed countries had paid attention to community health services and medical home services earlier [9]. Nowadays, with the acceleration of the aging population and consequent increase in the social security burden, many developing countries have started to focus more on the establishment of PHC as well [10]. Many governments have set out to improve the quality of community health services, with some success [11,12,13]. However, improvements in quality did not seem to have led to an increase in the utilization of community health services [14]. Due to traditional healthcare habits and more trust in large hospitals, Chinese residents still prefer to seek treatment in large hospitals [15,16]. How to change residents’ healthcare habits and increase the use of community health services is an important issue that needs to be tackled [17].

The COVID-19 pandemic has a significant impact on the political, economic, and social framework worldwide, especially in China [18,19]. China’s pandemic prevention and control policies are stricter and longer-lasting than other countries worldwide [20,21]. From the beginning of 2020 to the present, many aspects of people’s lives have changed significantly due to the pandemic, and healthcare is one of the key aspects [22,23]. 

On the supply side, pandemic prevention and control occupied a portion of healthcare resources, thereby impacting the overall supply of healthcare resources [24,25]. On the demand side, the psychology of patient access to care has changed, as hospitals were often perceived as places with a higher risk of contracting the virus [26]. As a matter of fact, prevention and control policies objectively increased the cost of patient access (both financial and time cost) and the unpredictability of the future access environment [27]. 

In these years, several studies focused on the impact of the COVID-19 pandemic on healthcare behavior. One study from the United States reported that 31.5% of U.S. adults discontinued routine medical care during the pandemic [28]. Studies have also shown that this avoidance of routine visits was more prevalent among those with underlying medical conditions [26,28]. Both at the patient and provider levels, the COVID-19 pandemic was likely to cause temporary interruptions in routine or non-emergency medical care access [29]. In response, methods have also been provided to help hospitals better allocate resources between COVID-19 and non-COVID-19 units [27].

However, the COVID-19 pandemic also has far-reaching implications for health systems and health services at other scales, some of which have not yet been investigated. While the COVID-19 pandemic has brought significant challenges to healthcare, it has also brought opportunities for change and innovation [30]. While some researchers pointed out the importance of improving the tiered healthcare system during the COVID-19 pandemic [31], others also reveal that confirmed the important role played by community involvement in the fight against the pandemic [32]. In developing countries with weakly developed PHC systems, the COVID-19 pandemic may provide an opportunity to strengthen the PHC at the community scale, i.e., in community health services. Therefore, the impact of COVID-19 on community health services needs to be evaluated.

Here, we examine the impact of COVID-19 on the utilization of community health services through data from Beijing, China. Specifically, community health centers were chosen as the specific embodiment of community health services. This study tested whether the COVID-19 pandemic or the various resulting policy measures promoted the utilization of community health services using community-level data. In the post-pandemic era, it is vital to learn from experiences and lessons during the pandemic to further improve the allocation of healthcare resources and construct a fairer and more efficient PHC system.

## 2. Methods

### 2.1. Data and Sample

The data were collected from the Community Health Service Center in Beijing, China, which contains all the patients who visited this community health center in a time interval from 1 January 2018 to 30 June 2021. The data were obtained in an encrypted and fully anonymized format, which provided information including the medical records of patients, the cost of healthcare services, and the personal characteristics of patients. 

In order to comprehensively investigate the effect of the COVID-19 pandemic on the use of community health services, we re-arranged the data into two sets: a time-series data set and a panel data set. The time-series data set includes variables such as the healthcare cost of all patients and its sub-category cost, the number of patient visits in each month from January 2018 to May 2021. Cases from all patients were taken into account. The panel data set contains all patients with chronic diseases in the same period of time. This study included 252,223 cases from 13,010 patients with chronic diseases.

### 2.2. Analytical Framework

The focal question of this study was whether the utilization of community health services is affected by the COVID-19 pandemic. This question relates to changes in the supply and demand of healthcare resources, which may have a long-term impact on residents’ health service utilization behaviors. This paper examines two aspects: the number of visits to community health centers before and after the occurrence of COVID-19 and the cost of healthcare. Changes in the number of patients reflected the macro-level impact of the epidemic on community health service utilization, while changes in healthcare costs revealed the micro-level impact. To test this impact, this paper analyzed using time-series data and panel dataset, respectively, to ensure the robustness of the findings.

In this paper, it was assumed that the COVID-19 pandemic affected the utilization of community health services mainly by changing the supply and access patterns of healthcare resources in society. The supply side of healthcare resources was affected by policy regulation, medical staff deployment, and public health measures during the pandemic, while the demand side was affected by pandemic risk perception, government propaganda, and health behavior changes. Based on the Anderson Model of Health Service Utilization (BMHSU), this paper proposed that the COVID-19 pandemic is more likely to affect the healthcare utilization behavior of the population by altering the enabling resources of health services. The BMHSU model emphasizes that the use of health services is influenced by predisposing characteristics, enabling resources, and need [33]. In the context of the COVID-19 pandemic, enabling resources for health services (e.g., healthcare accessibility and convenience of care) changed significantly, which in turn influenced patterns of community healthcare utilization.

**Figure 1:**
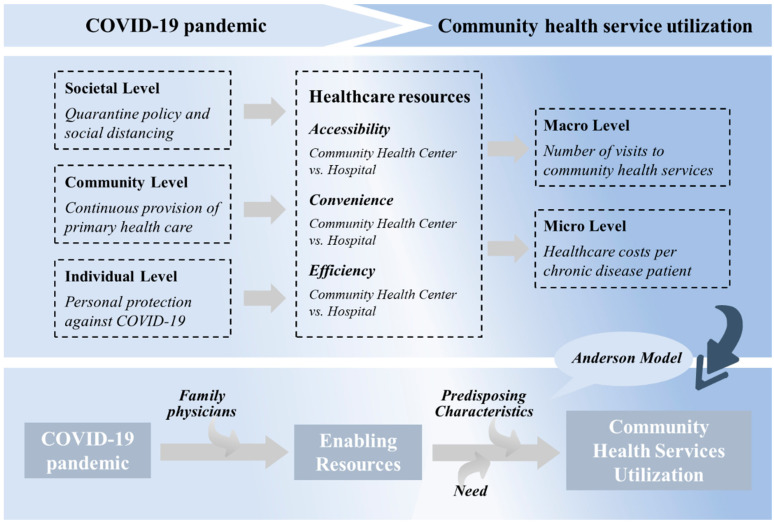
Analytical framework for the COVID-19 pandemic and community health service utilization.

In the analytic framework (Figure 1), the COVID-19 pandemic had a direct impact on healthcare at three levels: societal, community, and individual. First, at the societal level, the government implemented a series of pandemic prevention policies, such as travel restrictions, the prioritization of healthcare resources for pandemic prevention and control, and the promotion of telemedicine, which directly affected the mobility of healthcare resources. Second, at the community level, large hospitals have reduced general outpatient services due to epidemic prevention and control measures, making community health centers the main healthcare option for residents. In addition, community healthcare organizations also faced problems such as a shortage of medical supplies and difficulties with staff deployment, which affected their healthcare supply capacity. Finally, at the individual level, the epidemic led to changes in residents’ healthcare demand and healthcare-seeking behavior. On the one hand, some patients reduced the number of visits for non-acute illnesses due to concerns about cross-infection; on the other hand, the pandemic may have exacerbated uncertainty in the management of chronic illnesses, causing some patients to increase their reliance on community healthcare resources.

The COVID-19 pandemic led to sudden changes in the accessibility, convenience, and efficiency of healthcare resources, which in turn affected the utilization structure of community healthcare services. During the pandemic, healthcare resources from large hospitals were prioritized for pandemic prevention and control, making it difficult for ordinary patients to access routine healthcare services, and community healthcare became the primary choice for patients. As a result, at the macro-level, the number of patients attending community health centers may change, while at the micro-level, the medical costs per patient may be affected by changes in the supply and demand structure. In addition, this paper includes family physician contracting services as a key variable in the analytical framework to examine its role in community healthcare utilization during the epidemic. The family physician contracting service is an important model for integrating community healthcare resources, and the epidemic may have prompted it to play further roles, such as reducing unnecessary outings of patients through remote consultation or online consultation and enhancing the overall efficiency of community healthcare utilization. 

### 2.3. Variables

#### 2.3.1. Dependent Variables

As mentioned above, both the number of patient visits and healthcare costs may capture the utilization of community health services. As such, in the time series analysis, we took the cost of all patients together with sub-grouped fees of drugs, treatments, inspections, and materials in the community healthcare center as dependent variables, respectively. In addition, we also used both monthly patient visits and healthcare cost per capita as dependent variables. In the panel data analysis, the healthcare cost of each individual patient with chronic diseases was used as a dependent variable.

#### 2.3.2. Independent Variable: Identifying the COVID-19 Pandemic

A dummy variable, *T*, was set to identify the occurrence of COVID-19. *T* was set to 1 from 24 January 2020 and onwards, at which point the Chinese government activated a first-level public health emergency response. Prior to this time point, *T* was set to 0.

#### 2.3.3. Control Variables

The control variables in the panel data analysis were selected in line with the Anderson model. At the level of need, the diagnosis of patients with chronic diseases was taken into consideration to this end. Five dummy variables (hypertension, diabetics, CHD, CVD, and CRD) were set to distinguish between chronic disease categories. Among them, CHD indicates coronary heart disease, CVD stands for cerebrovascular disease, and CRD indicates a chronic respiratory disease. Together with hypertension and diabetes, we identified five types of chronic diseases. If these types of diseases were present in a patient’s diagnosis, the corresponding variable was set to 1. Otherwise, it was 0. Complications were used to distinguish between patients with complicated diseases or not. Complications were defined as cases where a patient’s diagnosis included not only a primary disease but also a secondary condition that resulted from or was directly caused by the primary disease, impacting the patient’s health or treatment outcome. At the level of enabling resources, family physician contracting and payment types were used for this purpose. For those who had contracted a family physician before treatment, the variable contracted was equal to 1. Payment types included commercial insurance, public healthcare, and self-payment. Self-payment was used as a control group to test the effect of insurance and the public. At the level of predisposing characteristics, the age and gender of patients were included. The variables system is shown in Table 1 below.

### 2.4. Models

#### 2.4.1. Time Series and Generalized Linear Models (GLMs)

Monthly healthcare data are normally affected by seasonal effects. Therefore, the seasonal effects of variables were tested in this study. With January taken as the reference time, the variables were regressed on the constants and the dummy variables from February to December, respectively. Table 2 shows the regression results.

**Figure 2:**
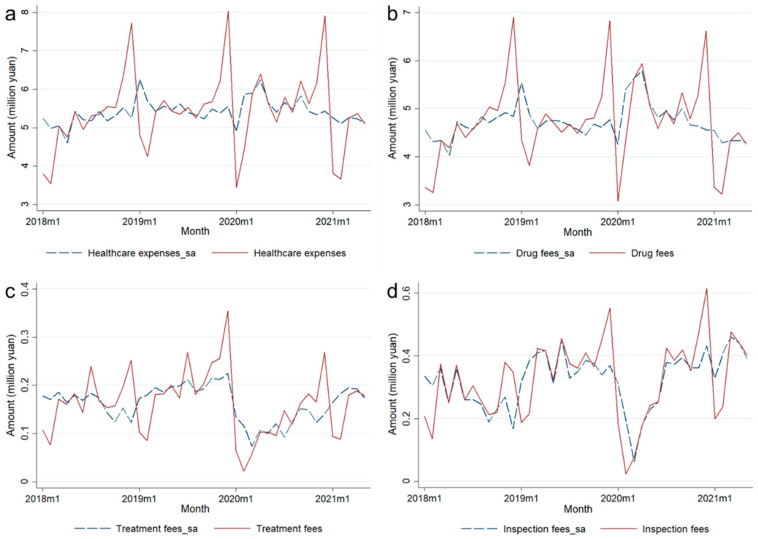
Data comparison of variables before and after seasonal adjustment. (**a**) Seasonal adjustment of total healthcare cost; (**b**) seasonal adjustment of drug fees; (**c**) seasonal adjustment of treatment fees; (**d**) seasonal adjustment of inspection fees.

A seasonal adjustment of the time series was performed using a regression method. The seasonal fluctuation characteristics of data on healthcare costs were reduced after the seasonal adjustment. In contrast, the seasonal adjustment effects of the *total healthcare cost* and *drug fees* were more evident than the other two variables (Figure 2). On the other hand, no seasonal adjustment was required for *material fees* or *healthcare costs per capita*.

In the time-series analysis, a generalized linear model (GLM) based on the seasonally adjusted data was applied, as follows:

(1) $${Utilization}_{ij}=\alpha_{j}+\beta_{j}T_{ij}+\varepsilon_{ij}\;\;\;\;\;\;\;\;\;$$

In Equation (1), ${Utilization}_{ij}$ represents the relevant dependent variables that captured the community health service utilization (including the *total healthcare cost*, *drug fees*, *treatment fees*, *inspection fees*, *material fees*, *patient visits*, and *healthcare costs per capita*). $T_{ij}$ is a dummy variable to distinguish the COVID-19 pandemic. The time-series model was constructed to verify whether the COVID-19 pandemic had a strong impact on community health service utilization. The GLM accounts for non-normality and heteroskedasticity in healthcare utilization data, ensuring flexible estimation.

#### 2.4.2. Panel Data and Two-Way Fixed-Effect Models (FE_TW)

The Two-Way Fixed Effects Model (FE_TW) controls for time-invariant heterogeneity and period-specific shocks, isolating the true impact of COVID-19. The model employed for the panel data analysis is as follows:

(2) $$\begin{array}{cc} {Expenses}_{it}=\beta_{0} & +\beta_{1}T_{it}+\beta_{2}{Contracted}_{it}+\beta_{3}{T_{it}\times Contracted}_{it}+\beta_{4}{Complications}_{it}+\beta_{5}{Hypertension}_{it}\\ & +\beta_{6}{Diabetics}_{it}+\beta_{7}{CHD}_{it}+\beta_{8}{CVD}_{it}+\beta_{9}{CRD}_{it}+\beta_{10}{Insurance}_{it}+\beta_{11}{Public}_{it}\\ & +{Individual}_{i}+{Month}_{t}+\varepsilon_{it} \end{array}$$

In Equation (1), ${Expenses}_{it}$ represents the healthcare cost of a visit to the community health center, $T_{it}$ is a dummy variable to distinguish the COVID-19 pandemic, and ${Contracted}_{it}$ represents information on the patient’s family physician contracting, which is 1 when a patient has contracted with a family physician and 0 otherwise. The interaction term ${T_{it}*Contracted}_{it}$ was also added to the regression equation to test the mixed effect of the COVID-19 pandemic and the family physician on healthcare costs. ${Complications}_{it}$, ${Hypertension}_{it}$, ${Diabetics}_{it}$, ${CHD}_{it}$, ${CVD}_{it}$, and ${CRD}_{it}$ represent information on the patient’s disease diagnosis. The control variables ${Insurance}_{it}$ and ${Public}_{it}$ record the patient’s payment type for each transaction. Coefficients $\beta_{1}$~$\beta_{11}$ reflect the effect of each variable on healthcare cost, ${Individual}_{i}$ represents the individual fixed effect for each patient, and ${Month}_{t}$ represents the time-fixed effect of the month in which the case took place. $\varepsilon_{it}$ indicates the random disturbance term.

According to the Hausman test results, it is more appropriate to choose a fixed-effect model, rather than a random-effect one. Additionally, it is also proper to include time effects in the individual fixed-effect model by testing the joint significance of the monthly dummy variables.

In the two-way fixed-effect model, individual effects are already considered, so variables describing individual characteristics were not added. A mixed regression model using OLS estimation was also used for comparison, in which control variables representing individual characteristics were added. The corresponding regression model is as follows:

(3) $$\begin{array}{cc} {Expenses}_{it}=\beta_{0} & +\beta_{1}T_{it}+\beta_{2}{Contracted}_{it}+\beta_{3}{T_{it}\times Contracted}_{it}+\beta_{4}{Complications}_{it}+\beta_{5}{Hypertension}_{it}\\ & +\beta_{6}{Diabetics}_{it}+\beta_{7}{CHD}_{it}+\beta_{8}{CVD}_{it}+\beta_{9}{CRD}_{it}+\beta_{10}{Insurance}_{it}+\beta_{11}{Public}_{it}+\beta_{12}{Age}_{it}\\ & +\beta_{13}{Gender}_{it}+\varepsilon_{it} \end{array}$$

${Age}_{it}$ and ${Gender}_{it}$ represent the individual characteristics, and cluster-robust standard errors were used in the mixed regression model.

## 3. Results

### 3.1. Time-Series Analysis Results

Firstly, a monthly time-series analysis was conducted before and after the pandemic, including data on healthcare costs (total healthcare costs together with drug fees, treatment fees, inspection fees, and material fees) and monthly patient visits over a time interval from January 2019 to May 2021. Furthermore, the time-series analysis was also respectively repeated for monthly patient visits and monthly healthcare costs per capita, from January 2018 to May 2021.

#### 3.1.1. Results of Descriptive Statistics

**Figure 3:**
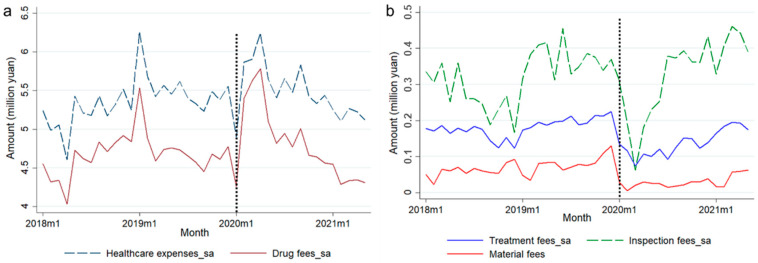
Trends in healthcare costs at the community level from Jan 2018 to May 2021. (**a**) Trends in total healthcare cost and drug fees (seasonal adjustment data are shown) over time; (**b**) trends in treatment fees, inspection fees, and material fees (seasonal adjustment data are shown for treatment fees and inspection fees) over time.

Table 3 shows the descriptive statistics for each variable, and Figure 3 presents fluctuations in five variables of healthcare costs. Among them, total healthcare cost and drug fees had a highly consistent monthly fluctuation trend since drug fees consist of a main component of the healthcare cost in community health services (Figure 3a). The fluctuation trends in treatment fees and material fees were also relatively similar due to the fact that material fees are primarily incurred during the course of treatment (Figure 3b). As can be roughly observed in the figure, both of them showed a certain decline after January 2020 as COVID-19 broke out. For total healthcare costs, drug fees, and inspection fees, there were no significant differences in their values before and after the COVID-19 outbreak.

**Figure 4:**
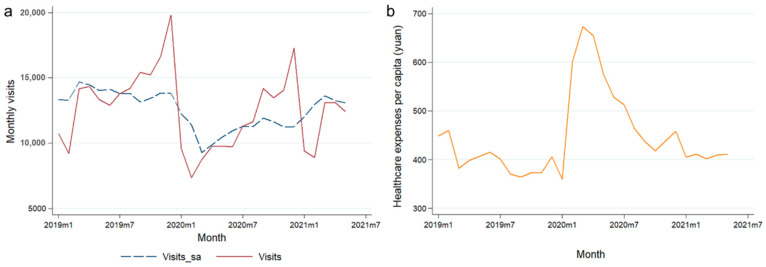
Trends in monthly visits and healthcare costs per capita at the community level from Jan 2019 to May 2021. (**a**) Trends in monthly visits (both seasonal adjustment data and raw data are shown) over time; (**b**) trends in healthcare costs per capita over time.

Trends in monthly visits and healthcare costs per capita are shown in Figure 4. With January 2020, the month of the COVID-19 outbreak, taken as a demarcation point, the number of monthly visits showed a trend of sharp decline in a short period of time, followed by a gradual fluctuation upward. In May 2021, the cut-off date for data collection, the visits had not fully recovered to their pre-outbreak level. The trend of monthly healthcare costs per capita was the opposite, showing a short sharp increase, followed by a gradual fluctuation downward after January 2020. 

**Figure 5:**
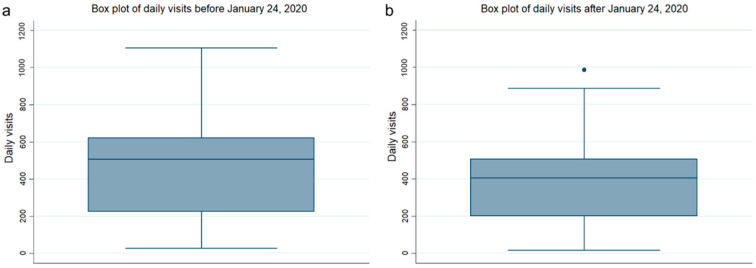
Box plot of daily visits at the community level. (**a**) Box plot of daily visits at the community level before 24 January 2020; (**b**) box plot of daily visits at the community level after 24 January 2020.

The daily visits to the community health center from 1 January 2019 to 31 May 2021 were also compiled (Figure 5). With the time point of 24 January 2020 taken as a demarcation point, the statistics were divided into two parts to draw box plots. Overall, daily visits decreased after the COVID-19 outbreak, consistent with the trend reflected in the monthly time-series data in Figure 4.

#### 3.1.2. Results of GLM

Model (1) was regressed using the seasonally adjusted data for all variables except medical material fees (Table 3 and Table 4). 

In Table 4, the results show that, after the COVID-19 outbreak, treatment fees and medical material fees decreased significantly by about 43,000 yuan and 40,000 yuan, respectively. Total healthcare costs, drug fees, and inspection fees were not significantly affected by the COVID-19 pandemic, which is consistent with the results observed in Figure 3. 

In Table 5, monthly visits decreased significantly after the outbreak, by about 2174; the monthly healthcare costs per capita increased significantly after the outbreak, by 80.054 yuan. For comparison, the change in total healthcare cost generated via community health service visits was also tested during this time interval, but it did not vary significantly before and after the outbreak, being consistent with the regression results in Table 3.

In summary, the statistical results of the monthly time series show that the COVID-19 pandemic significantly reduced visits to community health services and significantly increased the per capita healthcare costs of patients. With the combination of these two outcomes, the overall scale of healthcare costs did not change significantly in this period of time.

### 3.2. Panel Data Analysis Results

#### 3.2.1. Results of Descriptive Statistics and One-Way ANOVA

First, the panel data were divided into two groups based on whether the COVID-19 outbreak occurred. Descriptive statistics and a one-way analysis of variance (ANOVA) were conducted for each variable (Table 6). In this case, the median and interquartile range were reported for numeric variables, and the number and percentage of samples taking a value of 1 were reported for dummy variables indicating classification. In the one-way ANOVA, the F-statistic and *p*-value of the difference between the test groups were also reported.

The one-way ANOVA results show a significant difference in the healthcare cost of a single visit for patients with chronic diseases before and after the COVID-19 outbreak, with median values of 396.48 yuan and 435.16 yuan, respectively. For the family physician contracting variable, the proportion of contracted patients in the total sample increased from 75.42% to 79.02% after the outbreak, with significant between-group differences. Among the five diseases, the proportion of patients with these diseases increased after the outbreak, except for patients with chronic respiratory diseases. A similar increase in the proportion of complications occurred, with significant differences between groups. Notably, the decrease in the proportion of patients with chronic respiratory diseases is likely because patients with such diseases were restricted from visiting community health centers during the COVID-19 pandemic. These patients were often asked to go to specific hospitals.

**Figure 6:**
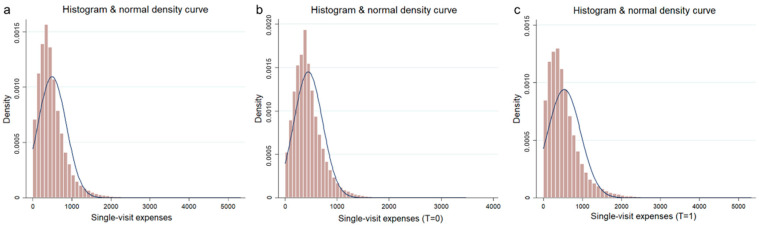
Histogram and normal density curve of healthcare costs. (**a**) Histogram and normal density curve of healthcare costs for total samples; (**b**) histogram and normal density curve of healthcare costs for samples before 24 January 2020; (**c**) histogram and normal density curve of healthcare costs for samples after 24 January 2020.

Furthermore, histograms and normal distribution density curves of single-visit healthcare costs were analyzed (Figure 6). Before the COVID-19 outbreak, healthcare costs were more centrally distributed. In contrast, after the outbreak, healthcare costs were distributed in a more dispersed manner, and the probability of healthcare costs exceeding 1000 yuan had increased compared to the previous period.

The descriptive statistics and one-way ANOVA depict the overall picture within the two sample groups and the differences between the groups. This analysis provided complementary information for the subsequent regression analysis.

#### 3.2.2. Results of FE_TW

A two-way fixed-effects model was used to test the effects of the COVID-19 pandemic, controlling for both time and individual effects. A difference-in-difference (DID) model was also used to test the implementation effect of the family physician contracting policy during the COVID-19 pandemic by constructing the variable of T*contracted. 

A stepwise regression method was used to include control variables in the model sequentially. Models 3, 6, and 9 used two-way fixed effects models. Model 10 used mixed regression and included all independent and control variables presented in this study. Since individual characteristic variables were not captured for all samples, Model 10 contained regression results for only 185,544 data.

The regression results show that, during the COVID-19 pandemic, the single-visit healthcare costs of patients with chronic diseases increased significantly (Table 7). In the three two-way fixed effects models, the healthcare costs of patients with chronic diseases and family physicians were significantly lower than those of non-contracted patients. During the pandemic, the cost-control effect of the family physician contracting policy was seemingly pronounced compared to the previous one.

## 4. Discussion

This paper has examined the possible effects of the COVID-19 pandemic on community health service utilization by employing micro-oriented patient data at the community level in Beijing, China. First of all, the descriptive statistics and regressive outcomes of the time-series data set indicated that there was a significant reduction in patient visits to community health centers during the COVID-19 pandemic. Secondly, the results of the two-way fixed effects model based on the panel data set suggested that the COVID-19 pandemic significantly raised the healthcare cost of a single visit for patients with chronic diseases. Finally, the results of evaluating the economic benefits of family physician contracting before and after the pandemic showed that the cost-control effect of family physician contracting increased during the pandemic.

**Figure 7:**
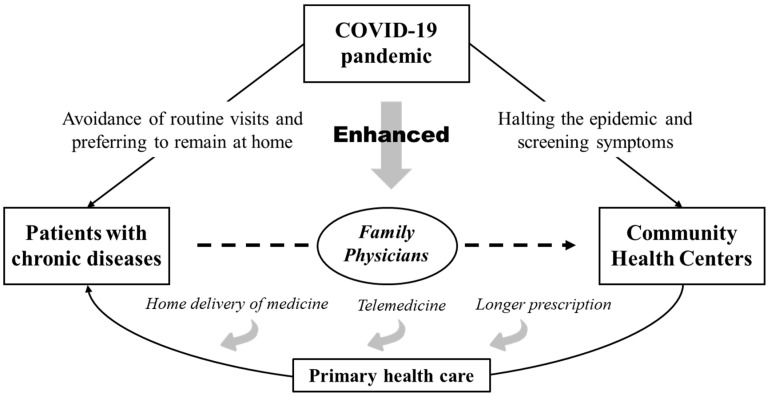
The pattern of providing health services to patients with chronic diseases at community health centers in Beijing, China, during COVID-19.

In summary, we compiled how community health centers in Beijing provided health services to patients with chronic diseases during COVID-19 (Figure 7).

### 4.1. The Avoidance of Routine Visits

From both the descriptive statistics and regression results, this study observed a decline in community health center visits during the COVID-19 pandemic. The avoidance of routine visits due to the pandemic has been previously identified in the literature at the hospital level [26,28,34], and this paper suggests that the decline also occurred at the community level.

In addition to the decrease in the number of patients, a disruption in routine care was also associated with a significant decrease in the total amount of treatments and medical materials. During COVID-19, non-essential and non-emergency treatments (e.g., surgery, etc.) were postponed or canceled. As a result of the social distancing, there were also patients who preferred a less restricted form of treatment with medications. This result was also found through similar studies in other countries. Researchers have found lower use of primary care and emergency departments, as well as the potential impact of the pandemic on some diseases such as CVDs [35].

Strategies related to COVID-19 control require healthcare providers to screen patients and healthcare workers for symptoms [36]. Initiatives such as wearing masks, social distancing, and staying at home also increased patients’ avoidance of routine visits [36]. Psychosocial elements have changed, while telemedicine and remote monitoring have evolved rapidly in this process [37,38]. The COVID-19 pandemic has changed the pattern of patient access to societal healthcare resources over the past three years, thus influencing the way healthcare resources are provided.

Policymakers may consider the following policy recommendations. First, they could strengthen the infrastructure of community healthcare resources and the system of hierarchical diagnosis and treatment. Studies have shown that community health centers become the main medical choice for patients during epidemics, so the government should further enhance the service capacity of community medical institutions. In addition, the tiered diagnosis and treatment system should be strengthened to optimize the flow of care for patients, and non-acute patients should be guided to give priority to community healthcare institutions so as to reduce the pressure on large-scale hospitals and enhance the service effectiveness of the primary healthcare system.

### 4.2. Increase in Healthcare Costs

Community health centers mainly provide basic healthcare services for patients with chronic diseases who may obtain medical resources such as medications conveniently and efficiently [39]. The healthcare costs of patients who visit community health service centers are in line with their utilization of community health services. The increase in healthcare costs found in this paper may reflect that the pandemic expanded the use of community health services.

During COVID-19, the Chinese government introduced measures to safeguard the needs of patients with chronic diseases for routine healthcare. Medical institutions are encouraged to provide longer prescription services. The healthcare insurance administration implemented a “long prescription” reimbursement policy during the pandemic era. Community health centers also increased the amount of medication in a single prescription and reduced the number of patient visits for doctors to dispense medication so as to protect the long-term medication needs of patients with chronic diseases. Indeed, these measures alter the allocation of healthcare resources on the supply side.

Most patients with chronic diseases require long-term medication, and a large portion of the healthcare costs they incur at community health centers are made up of medicine fees. While the pandemic has objectively changed medication access patterns, more patients are choosing to visit community healthcare centers, rather than large hospitals for their prescriptions. Additionally, the inconvenience of travel and the uncertainty of healthcare availability due to the pandemic restrictions also made patients tend to increase the number of single prescriptions. The combination of these factors resulted in a significant increase in the healthcare cost for a single visit during COVID-19.

### 4.3. Family Physicians During the Pandemic

Community health services are underutilized in most developing countries. Nevertheless, an outbreak of COVID-19 appears to provide an opportunity to reallocate healthcare resources by changing patients’ access habits. In this process, it is notable that family physicians are playing a vital role.

Family physicians are an essential part of PHCs, which function as an important bridge for patients with chronic diseases to utilize community health services [40]. Building a family physician contracting system is also an important aspect of China’s healthcare reforms [41]. The existing study documents that family physicians improve the accessibility of healthcare during the pandemic [42]. The findings of this paper further verify that family physician contracting played a vital role in securing healthcare services for patients with chronic diseases after the COVID-19 outbreak.

Initially, policymakers assumed that family physicians act as “health gatekeepers” and “healthcare cost gatekeepers” for patients with chronic diseases [2,43]. This role was accentuated by the effect of the pandemic. Patients who have contracted with a family physician will have much lower costs and less uncertainty about the availability of future medical resources than those who have not. Contracted patients may also enjoy services such as telemedicine and the “home delivery of medicine” provided via community health centers during the pandemic in Beijing, China. Family physicians have become important guarantors of stable healthcare for patients during the pandemic time.

### 4.4. Future Directions

Although this study revealed the impact of the COVID-19 pandemic on the utilization of community healthcare services, certain limitations remain. The data of the study were mainly derived from Beijing, which may limit the external applicability of the findings. Because Beijing has relatively abundant healthcare resources and a well-developed community healthcare system, its healthcare utilization pattern during the epidemic may differ from that of other regions (e.g., small and medium-sized cities or rural areas with fewer healthcare resources). Therefore, future research could be expanded to different regions to examine the performance of community healthcare services under different healthcare systems and policy environments to enhance the generalizability of the study.

In the future, longitudinal studies can be used to continuously track the evolution of the utilization pattern of community healthcare services, such as whether the demand for healthcare returned to the pre-epidemic level in the latter part of the epidemic or whether new healthcare utilization habits were formed. In addition, policy experiments or natural experiments can be considered to analyze the long-term impact of different epidemic prevention and control policies (e.g., hierarchical diagnosis and treatment, health insurance payment adjustment) on community healthcare utilization so as to provide more targeted empirical support for policy formulation.

## 5. Conclusions

This paper aimed to investigate the linkage between the utilization of community health services and the prevalence of COVID-19 in China. Specifically, this study employed generalized linear models based on time-series data and two-way fixed-effect models based on panel data from a community center in Beijing to test whether the pandemic had a phenomenal effect on community healthcare services. In addition, we also used a difference-in-differences model to examine the mixed effects of the pandemic and family physician contracting. The empirical evidence suggested that, although the number of visits at the community level objectively declined due to the pandemic, it may be partially compensated for via an increase in per visit healthcare costs. That is, patients with chronic diseases who need long-term medication may secure drugs by reducing their number of visits and increasing their dosages of medication in a single prescription. During the pandemic, family physicians played an important role in connecting patients with community health centers. Family physicians can provide more reliable and stable long-term services for patients, reducing the uncertainty that the pandemic brings to patients’ routine care while cutting unnecessary healthcare costs. COVID-19 has objectively made contracting a family physician a reasonable and rational choice for patients with chronic diseases.

While COVID-19 plagues China’s healthcare system, it also poses a possibility for the development of community healthcare services. As a matter of fact, grassroots community services are playing a vital double roles in both containing the spread of the epidemic and safeguarding the regular needs of patients. China still has a long way to go to move from passive “hierarchical health care” to proactive “primary health care”, and COVID-19 certainly provides an opportunity to do so. It is crucial for Chinese policymakers to seize this chance to realign healthcare resources in order to enhance the well-being of the general public. The COVID-19 outbreak has led both policymakers and patients to revisit community healthcare centers to promote the utilization of available healthcare services. It is essential to improve primary healthcare to face off the surge of chronic disease complications, especially when some chronic diseases were under-treated and under-controlled during the pandemic. Therefore, the further enforcement and improvement of the primary healthcare system will be focal issues in the near future. This may be one of the invaluable lessons learned from the COVID-19 pandemic, and it may also be useful for other developing countries.

## Figures and Tables

**Table 1:** Variable system.

Variables	Description
Time series: dependent variables	
Total healthcare cost	Total healthcare cost at the community health center in a month (seasonally adjusted)
Drug fees	Total drug fees at the community health center in a month (seasonally adjusted)
Treatment fees	Total treatment fees at the community health center in a month (seasonally adjusted)
Inspection fees	Total inspection fees at the community health center in a month (seasonally adjusted)
Material fees	Total material fees at the community health center in a month
Patient visits	Sum of visits for patients at the community health center in a month (seasonally adjusted)
Healthcare costs per capita	Average healthcare cost for patients at the community health center in a month
Time series: independent variable	
T	Dummy variable to distinguish the COVID-19 pandemic (at or after January 2020 = 1, before January 2020 = 0)
Panel data: dependent variable	
Costs	Healthcare costs of patients for a visit to the community health center
Panel data: independent variable	
T	Dummy variable to distinguish COVID-19 pandemic (at or after 24 January 2020 = 1, before 24 January 2020 = 0)
Panel data: control variables	
Contracted	Whether the patients had contracted a family physician (contracted = 1, not contracted = 0)
Complications	Whether the patients with complications (with = 1, without = 0)
Hypertension	Whether the diagnosis with hypertension (with = 1, without = 0)
Diabetics	Whether the diagnosis with diabetics (with = 1, without = 0)
CHD	Whether the diagnosis with coronary heart disease (with = 1, without = 0)
CVD	Whether the diagnosis with cerebrovascular disease (with = 1, without = 0)
CRD	Whether the diagnosis with chronic respiratory diseases (with = 1, without = 0)
Insurance	Whether the patients paid with healthcare insurance (with = 1, without = 0)
Public	Whether the patients with public healthcare (with = 1, without = 0)
Age	The age of patients
Gender	The gender of patients (males = 1, females = 0)

**Table 2:** Regression results of healthcare cost on monthly dummy variables.

Healthcare Cost Variables	*F*	R-Squared	Results
Time: from January 2018 to May 2021			
Total healthcare cost	24.62 ***	0.9033	Seasonal adjustment required
Treatment fees	6.23 ***	0.7025	Seasonal adjustment required
Inspection fees	2.76 **	0.5116	Seasonal adjustment required
Material fees	1.35	0.3383	No seasonal adjustment required
Drug fees	12.13 ***	0.8215	Seasonal adjustment required
Time: from January 2019 to May 2021			
Patient visits	4.82 ***	0.7571	Seasonal adjustment required
Healthcare costs per capita	0.37	0.1913	No seasonal adjustment required

Note: * p<0.1, ** p<0.05, *** p<0.01; all standard errors are robust standard errors.

**Table 3:** Descriptive statistics of variables for time-series analysis.

Variables	Median/Frequency	IQR/Percentage (%)
Total healthcare cost	5,406,989	323,830
Drug fees	4,678,201	279,701
Treatment fees	173,325.3	53,296.83
Inspection fees	339,189.8	123,873.5
Material fees	55,764.1	42,490.89
Patient visits	13,099.63	2498
Healthcare costs per capita	411.30	58.93
T	17	41.46

Note: (1) seasonal adjustment data were used for total healthcare costs, drug fees, treatment fees, inspection fees, and patient visits; (2) non-normally distributed healthcare cost variables are expressed in the median and interquartile range, and T is expressed as a frequency and a percentage; (3) the unit of healthcare cost variables is yuan.

**Table 4:** Effects of the COVID-19 pandemic on four types of healthcare costs at the community level (time-series data from Jan 2018 to May 2021).

Dependent Variables (Unit: Million Yuan)	Independent Variable: T	
	Coefficients (95% CI)	Standard Errors
Total healthcare costs	0.110 (−0.094–0.315)	0.101
Drug fees	0.110 (−0.125–0.346)	0.116
Treatment fees	−0.043 *** (−0.062–−0.023)	0.010
Inspection fees	0.008 (−0.050–0.065)	0.028
Material fees	−0.040 *** (−0.053–−0.027)	0.006

Note: * p<0.1, ** p<0.05, *** p<0.01, all standard errors are robust standard errors.

**Table 5:** Effects of the COVID-19 pandemic on monthly visits and healthcare costs per capita at the community level (time-series data from Jan 2019 to May 2021).

Dependent Variables	Independent Variable: T	
	Coefficients (95% CI)	Standard Errors
Total healthcare cost	−0.066	0.113
Visits	−2173.507 ***	359.091
Healthcare costs per capita	80.054 ***	28.480

Note: (1) * p<0.1, ** p<0.05, *** p<0.01; all standard errors are robust standard errors. (2) For the data used in the regression, the unit of “total healthcare cost” is million yuan, and the unit of “healthcare costs per capita” is yuan.

**Table 6:** Descriptive statistics and one-way ANOVA based on the grouping of T.

Variables	T = 0	T = 1	*F*	*p*-Value
	(T: Dummy Variable to Distinguish COVID-19 Pandemic)			
Costs	396.48 (320.94)	435.16 (463.22)	3668.02	<0.001
Age	64.05 (13.52)	65.27 (13.64)	590.71	<0.001
Gender				
Male	42,075 (47.97%)	47,068 (48.09%)	0.24	0.622
Family physician				
Contracted	89,156 (75.42%)	105,889 (79.02%)	464.14	<0.001
Diagnosis				
Hypertension	56,255 (47.59%)	73,388 (54.76%)	1301.14	<0.001
Diabetics	34,489 (29.18%)	41,226 (30.76%)	75.42	<0.001
CHD	55,474 (46.93%)	73,785 (55.06%)	1673.51	<0.001
CVD	23,716 (20.06%)	30,563 (22.81%)	280.41	<0.001
CRD	23,317 (19.72%)	22,879 (17.07%)	295.60	<0.001
Complications	106,702 (90.26%)	124,370 (92.81%)	530.22	<0.001
Payment type				
Insurance	113,704 (96.19%)	128,730 (96.06%)	2.66	0.103
Public healthcare	2644 (2.24%)	3009 (2.25%)	0.02	0.883
Self	1495 (1.26%)	1746 (1.30%)	0.72	0.395

**Table 7:** Effects of the COVID-19 pandemic on healthcare costs at the community level (panel data from patients with chronic diseases).

Variables	1	2	3	4	5	6	7	8	9	10
	Single-Visit Healthcare Cost									
T	92.48 *** (4.59)	123.42 *** (4.97)	112.71 *** (4.93)	69.30 *** (4.43)	100.83 *** (4.86)	90.84 *** (4.82)	70.50 *** (4.42)	101.56 *** (4.88)	91.55 *** (4.84)	81.95 *** (6.40)
Family physician (Contracted = 1)	26.35 *** (4.02)	−67.23 *** (7.91)	−56.41 *** (7.94)	13.47 *** (3.47)	−65.80 *** (7.69)	−55.85 *** (7.71)	13.67 *** (3.35)	−65.28 *** (7.54)	−55.36 *** (7.57)	8.14 * (4.75)
T*Contracted	−7.49 (5.23)	−17.75 *** (5.60)	−15.59 *** (5.56)	−6.55 (5.03)	−20.66 *** (5.43)	−18.64 *** (5.39)	−7.58 (5.01)	−21.31 *** (5.45)	−19.27 *** (5.41)	−18.05 *** (6.92)
Control variable type 1: diagnosis of diseases										
Complications				63.18 *** (3.11)	30.54 *** (3.06)	31.41 *** (3.07)	59.85 *** (3.11)	30.50 *** (3.06)	31.37 *** (3.07)	54.51 *** (3.73)
Hypertension				81.90 *** (2.54)	85.79 *** (2.24)	83.62 *** (2.23)	80.80 *** (2.51)	85.77 *** (2.24)	83.60 *** (2.23)	81.34 *** (2.95)
Diabetics				153.04 *** (3.44)	135.62 *** (2.93)	134.83 *** (2.92)	153.19 *** (3.38)	135.66 *** (2.93)	134.88 *** (2.92)	156.47 *** (3.91)
CHD				115.46 *** (2.53)	93.56 *** (2.11)	92.65 *** (2.10)	114.09 *** (2.51)	93.54 *** (2.11)	92.64 *** (2.10)	112.27 *** (2.96)
CVD				204.75 *** (3.77)	167.73 *** (3.05)	167.60 *** (3.04)	203.13 *** (3.69)	167.65 *** (3.05)	167.53 *** (3.04)	203.30 *** (4.30)
CRD				76.48 *** (3.22)	56.54 *** (2.69)	55.85 *** (2.69)	75.20 *** (3.19)	56.53 *** (2.69)	55.85 *** (2.69)	74.04 *** (3.83)
Control variable type 2: payment type										
Insurance							106.26 *** (8.24)	4.84 (8.86)	6.62 (8.89)	110.78 *** (12.79)
Public healthcare							283.61 *** (17.77)	128.30 *** (42.28)	124.65 *** (42.43)	304.17 *** (22.42)
Control variable type 3: individual characteristics										
Gender										−5.54 (3.98)
Age										−0.21 (0.18)
Individual FE	No	Yes	Yes	No	Yes	Yes	No	Yes	Yes	No
Time FE	No	No	Yes	No	No	Yes	No	No	Yes	No
N_g	13,010	13,010	13,010	13,010	13,010	13,010	13,010	13,010	13,010	8010
N	252,223	252,223	252,223	252,223	252,223	252,223	252,223	252,223	252,223	185,544

Note: (1) * p<0.1, ** p<0.05, *** p<0.01. (2) Models 1–3 show the effects of the COVID-19 pandemic and family physician contracting on single-visit healthcare costs for patients with chronic diseases at the community level in China; control variables representing disease diagnosis were added to Model 4–6, control variables representing payment type were added to Model 7–9, and control variables representing individual characteristics were added to Model 10. (3) Models 3, 6, and 9 used two-way fixed effects.

## Data Availability

The data supporting the findings of this study are available from the corresponding authors upon reasonable request.
